# Is ageing becoming more active? Exploring cohort-wise changes in everyday time use among the older population in Sweden

**DOI:** 10.1007/s10433-021-00647-1

**Published:** 2021-08-17

**Authors:** Bertil Vilhelmson, Eva Thulin, Erik Elldér

**Affiliations:** Department of Economy and Society, Human Geography Unit, School of Business, Economics and Law, University of Gothenburg, Box 630, 405 30 Gothenburg, Sweden

**Keywords:** Time use, Cohort comparisons, Generational shifts, Productive activities, Older adults, Daily activities

## Abstract

The time older people spend on various daily activities is critical for their health and well-being. New generations of older adults are increasingly expected to participate in ‘active’ activities. We explore shifts in active time use among upcoming cohorts of older people in Sweden. Recognizing the diverging meanings associated with the active ageing concept, we develop a classification model comprising the spheres of work, social engagement, and active leisure. We observe differences in time use of the ‘older middle-aged’ (pre-retirement), ‘young old’, and ‘older old’ observed in 2000/2001 and 2010/2011. We draw on two cross sections of Swedish time-use survey data covering 120 activities related to people’s everyday lives. We measure between-cohort differences in mean time use and employ covariate analysis to control for the influence of group-wise changes in socio-demographics. Linear regression is used to explore social differentiation, e.g. the influence of gender. Comparisons between new and previous generations indicate substantial increases in overall active ageing activity: increases by 7 h per week among the older old and 3.5 h among the young old and older middle-aged. New generations spend more time on work, paid or unpaid, and leisure digital interaction; for some, this is counteracted by less free time spent on social engagement. The new generation of the older old group spends more time on outdoor activity and exercise. These time-use patterns are gendered and dependent on education, mainly due to changes in cohort composition.

## Introduction

Due to increasing life expectancy, people are expected to spend an increasingly larger proportion of their lives in ‘old age’ as pensioners. In some western countries, the time spent in retirement may even come to exceed the time spent in the active working life (Gauthier and Smeeding 2010). This development challenges ageing societies by increasing the burden on the healthcare and welfare services. Yet, it may also present new opportunities, since the conditions for ageing and retirement have improved remarkably in recent decades. New generations of older people are generally healthier, better off, and more capable of living active lives and positively engaging with the world than were previous generations (Katz 2000; WHO 2015; Principi et al. 2018; Skoog 2020). Today’s elders arguably no longer regard retirement as marking the inevitable disengagement from social roles, but as the emergence of a new phase—a ‘third age’ (Laslett 1989, 1994)—that brings unrestricted time, temporal autonomy, and latitude for new forms of engagement in and contribution to society (Bass 2000; Gilleard and Higgs 2002; Sabbath et al. 2016).

Theoretically, this coming of a third age in later life is relevant to the influential notion of ‘active ageing’, underlining that the continued participation of older people in society is crucial and necessary for both collective and individual reasons (Hinterlong et al. 2001; Sabbath et al. 2016; Zaidi and Howse 2017). Although in common use in policy, research, and practice, the active ageing concept lacks clarity regarding its interpretation, measurement, and outcome. Authors disagree in attempting to define the concept (Clarke and Warren 2007; Boudiny 2013). As widely conceived, active ageing refers to activities that have value for society—both economic (e.g. paid work, unpaid care work, and volunteering) and social (e.g. social networking and support)—as well as for individual health, well-being, and independent living (including active leisure). However defined, how people choose to use their *time* to engage in various activities in daily life is one prominent indicator of the development of active and potentially successful ageing (Sprod et al. 2015, 2017; Strazdins et al. 2016; Weir et al. 2018).

Whereas the benefits of active ageing are widely acknowledged and are expected to increase due to older people’s better health and capabilities, to what extent and in what forms upcoming generations of older people really engage in active ageing activities remain issues of concern. As noted (Katz 2000; Sabbath et al. 2016; Wanka 2019a, b; Kim 2019), changes in the time used for active ageing activities between and within generations are not well documented or understood. Most previous time-use studies are static (i.e. comparing older and younger people at one point in time), small in scale, or observe only a few activities, such as paid work or social pursuits, neglecting other time uses during the day, so there is a lack of comprehensive and dynamic approaches. This study helps empirically to fill this research gap. For this purpose, we also describe and develop the concept of ‘active ageing activities’.

### Aim and research questions

In this paper, we aim to explore generational shifts in overall time use for active ageing activities among consecutive cohorts of older adults. Drawing on repeated cross-sectional data from the Swedish time use surveys of 2000/2001 and 2010/2011, we explore how the daily time-use patterns and priorities of new generations of elders differ from those of previous generations. Central to this study is the belief that people’s regular activity patterns—i.e. their time spent on different activities and for different purposes—have crucial implications for individual health and well-being (Weir et al. 2018; Bauman et al. 2019).

We pose three research questions. *First*, do newer generations of older people spend more time on active ageing activities in different spheres of everyday life than did previous ones? Since active ageing is an ambiguous and contentious concept as regards content, we examine various interpretations of it. *Second*, are observed changes in active time use contingent on structural differences in cohort composition or on changing preferences? This means that we explore whether observed changes are solely because emerging cohorts differ in sociodemographic structure, or whether they also differ in individual behaviour and choice. *Third*, to what extent is active time use in later life socially differentiated as regards individual socioeconomic status, gender, and living region? We consider this in order to challenge the assumption of homogeneity attached to the third age and active ageing concepts and to highlight potential inequality.

Observably, given the cross-sectional nature of the data, our ambition is limited to comparing the characteristics differing between new and previous age cohorts of ‘older middle-aged’ (pre-retirement 55–64 years old), ‘young old’ (65–74 years old), and ‘older old’ (75–84 years old), respectively. Seeking, for example, to trace and separate the influences of age, period, and cohort (APC) would require longitudinal data (see e.g. Bell 2020).

## Theoretical approach and literature review

Theoretically, we depart from a time-use perspective, looking deeper into the temporalities of ageing. This perspective assumes that differences and changes in the conditions, opportunities, norms, and practices of ageing have first-level implications for the daily use of time (Gershuny 2000; Shove 2009; Michelson 2015; Vilhelmson et al. 2017). Direct changes in the time spent on different activities, in different contexts and with different levels of physical and cognitive activity, have further repercussions at the individual level in terms of health and well-being (Bauman et al. 2019; Weir et al. 2018; Jun and Suhs 2019) and at the societal level in terms of economic productivity and dependency on the social care system (Gauthier and Smeeding 2010; Sabbath et al. 2016). Time-use theory concentrates on the dynamic relationships between daily activities, given that time is a limited resource making up 24 h of a day (Gershuny 2003; Schwanen and Ziegler 2011). These relationships include the basic fluctuations, reorganizations, and priority shifts in daily activities that come with major life transitions such as retirement.

Regarding established social theories of ageing, various assumptions can be made about time-use shifts and reorganizations, as people’s roles, relationships, and engagements develop as they age and retire (Marcum 2013). In short, these theories have partly conflicting implications for how people prioritize and modify their time use in different spheres of everyday life. While the classical theory of disengagement (Cumming et al. 1960; Hochschild 1975) portrays ageing as the successive withdrawal from ‘active’ activity spheres (e.g. work, parenting, and social networking), other theories treat retirement as a period of sustained involvement and engagement. Activity theory (Havighurst 1961) maintains that while time use changes, the personal utility of performing activities does not, suggesting that people will continue to engage in socially rewarding activities. Continuity theory (Atchley 2001) maintains that people in retirement transitions essentially strive for consistency in lifestyles and core values and, at some level of abstraction, also in their daily use of time, for example, replacing time for paid work with volunteering, or the parenting role with time spent with grandchildren.

These social theories of ageing are criticized for being simplistic, viewing ageing as a universal experience that remains largely invariant over generations (Casalanti 1996; Principi et al. 2018; Wanka 2020). Focusing primarily on psychosocial components, they foster a limited understanding of how structural differences and changes in society influence time use in later life (Gershuny 2000; Gilleard and Higgs 2002, 2007). In particular, critics note that the conditions of ageing and retirement have changed drastically over time, and that newer generations of older people (e.g. ‘the baby boomers’) are generally healthier, better off, more educated, more gender equal, have more qualified jobs, and have better access to means of communication (e.g. cars and the Internet). This development was famously conceptualized as the emergence of a ‘third age’ (Laslett 1994), suggesting that ageing and retirement, rather than marked by disengagement and separation from social roles, offer scope and time for a new and active phase of life and for continued participation in society (Katz 2000; Gilleard and Higgs 2002; Sabbath et al. 2016; Wanka 2019a). This revises the conception of old age, giving it positive meaning, as older people are portrayed as active and autonomous individuals who are architects of their own future (Wanka 2019b). They maintain skills, competences, and preferences during retirement, and continue to lead active and independent lives, being part of and making valuable contributions to society (Schwanen and Ziegler 2011; Principi et al. 2018).

‘Third age thinking’ is connected to a number of slightly divergent approaches articulated over the last few decades. These approaches include ‘active ageing’ (Walker 2002; WHO 2002, 2015), ‘healthy ageing’ (WHO 1990), ‘successful ageing’ (Rowe and Kahn 1997, 2015), and ‘productive ageing’ (Butler and Gleason 1985; Hinterlong et al. 2001), all of which endorse active pursuits and life engagements as key components in sustaining good health and physical and mental capacity in old age. These approaches represent a shift away ‘from seeing old age as a condition that requires support and assistance to a process that we can evaluate as going more or less well’ (Zaidi and Howse 2017: 2). In particular, the ‘active ageing’ conceptualization has been influential in research and policymaking (Foster and Walker 2014; Walker and Zaidi 2016; Zaidi and Howse 2017).

However, while the benefits of active ageing for individuals and society are widely acknowledged, there is no consensus among researchers on how to identify, measure, and evaluate the concept in terms of concrete daily activities (Boudiny and Mortelmans 2011; Boudiny 2013). An important distinction can be made between studies using a society-oriented, economic definition concentrating on a few work-related (‘productive’) activities, and studies applying an individual-centred, sociological interpretation that emphasizes the well-being of individuals from a broader activity perspective. The narrower definition of productive activity proposed by, for example, Gonzales et al. (2015), includes time spent on paid work valued by the market and similar work activities valued more implicitly, such as caregiving for others (otherwise performed by paid staff) and voluntary work in organizations that produce social benefits. Sabbath et al. (2016) extended the meaning of productive activity by also including informal social interaction and support in their concept of ‘productive engagements’. This concept refers to emotional, appraisal, and instrumental transactions in personal social networks, relationships, and contacts. Still broader interpretations of active ageing are more person-centred and include a wide range of activities believed to support the individual’s ability to maintain health and independent living (Butler and Schecher 1995; Bass 2000; Chatzitheochari and Arber 2011; Kim 2019). Kim (2019), for example, argued that physically and mentally demanding leisure activities such as exercise, outdoor activity, and reading should be considered, as well as housework, maintenance work, self-management, and maintaining appearance. These activities are indirectly also beneficial at the societal level by reducing healthcare and community service expenditures.

Whereas the active ageing of ‘third agers’ is expected and helped to develop over time, given the better capabilities and prospects of new generations of older adults, to our knowledge very few studies have scrutinized generational shifts in time spent on active ageing activities. Gauthier and Smeeding (2010) examined changes in cohorts of older adults between 1960 and 1990 in the USA, UK, and Netherlands. Their study found a considerable decrease in time spent on paid work, particularly among older middle-aged men, confirming a well-known trend towards early retirement and fewer hours worked in the studied ‘pre-third age’ period. Yet, among older middle-aged women there was a slight increase in time spent on paid work. There was no evidence that lower levels of paid work were counterbalanced by more time spent on active ageing activities such as unpaid work, volunteering, or social support. Time was mainly reallocated to housework and leisure activities (both passive and active). In a more recent study, Jun and Suhs (2019) examined changes in activity among cohorts of older adults (aged 65 years and over) between 1985 and 2015 in the UK, still finding no overall increase in the levels of paid work. Time spent on paid work decreased among older men, while it increased slightly among women. However, there were indications of enhanced ‘productivity’ in other regards, as time spent on unpaid work increased considerably. Also, time spent on household chores and active leisure activities increased. Notably, there was a considerable decrease in time spent on social support activities. In a small longitudinal study of how daily time use changed across the retirement transition among a group of retirees in Australia, Sprod et al. (2017) found that paid working time was replaced with only a small amount of time spent in physical activity. Less time was spent on active transport, while more time was spent on sedentary activities (e.g. watching television) and regular household tasks.

Adding to this, a few studies have examined differences in time use among various groups of older people, casting light on social inequalities in active ageing and temporality. Chatzitheochari and Arber (2011), using data from the UK Time Use Survey of the year 2000, found that only a small minority of British retirees (aged 65 years and over) at that time regularly engaged in active leisure pursuits in a typical week. Instead, older people mostly participated in indoor activities, such as watching television and other passive types of mass media consumption. A gender difference was found, with men more likely than women to engage in active leisure activities, even after adjusting for gender differences in socioeconomic conditions and health. Active leisure was more likely among healthy men of a privileged educational and occupational background. Similar results were obtained by Sabbath et al. (2016) in a study of a group of French retirees, 60–74 years old. The amounts of time spent on paid work, unpaid work, and social support were higher among groups of high socioeconomic status, suggesting that such time use should increase over time, as new generations are generally expected to be wealthier. They also found clear gender differences, with women being less likely to participate in paid work, volunteering, and community activity, and men being less likely to participate in caregiving and social support. Similar patterns regarding active ageing and gender were found by Kim (2019) in a time-use study of 60–74-year-old South Koreans and by Wanka (2020) in her exploration of data from the German Time Use Survey 2012/2013.

In sum, the few existing dynamic studies of daily time use, performed in various contexts, have so far not given a clear picture of the extent to which ageing is currently becoming more active, as expected from the active ageing and third age theories, as regards work, broader social engagement, and active leisure. The static studies indicate that active time use patterns are diverse, gendered, and differentiated as regards socioeconomic, professional, and educational status. Knowledge is still limited when it comes to how consecutive generations of older people, with better capabilities and opportunities, actually change their comprehensive activity patterns and participation in society.

### Spheres of daily activities related to active ageing

**Fig. 1:**
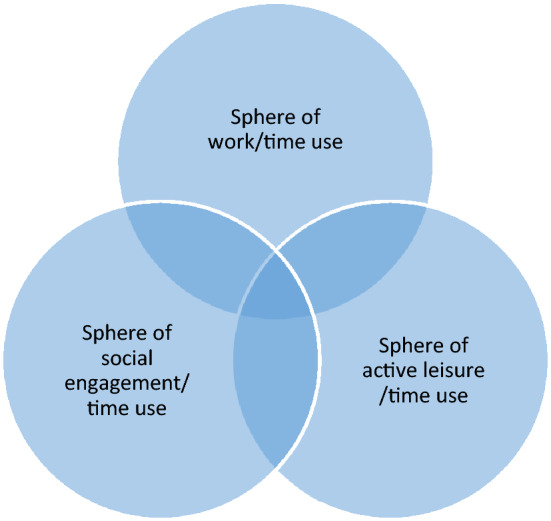
Spheres of active ageing activities and time uses considered important for individual health and well-being

As emphasized above, previous studies have revealed that active ageing is a diverse and contested concept as regards what daily activities should be accounted for. We therefore advance a time-use classification model drawing on the multiple meanings attached to the active ageing framework (and adjacent conceptualizations) presented in the literature. As key defining criteria, we concentrate on activities that involve the individual in the wider economy, social life, and physical and mental activation. Accordingly, we recognize activities within three spheres of time use in everyday life: work-related activities, social engagement, and active leisure (Fig. 1).

The first sphere concerns productive work activities understood by, for example, Bass et al. (1993) and Gonzales et al. (2015), as any activities, whether paid or unpaid, undertaken by an older individual that produce goods or services for society. This comprises paid employment (including self-employment), caregiving for an adult or child outside the household, and volunteering for organizations creating social goods. The second sphere comprises social engagement activities*.* In line with, for example, Morrow-Howell et al. (2012) and Sabbath et al. (2016), this refers to individual participation in the activities of a social group of friends, relatives outside the household, or neighbours to reinforce social capital. Key elements of active social engagement include activity (i.e. doing something), interaction (involving at least two people), and social exchange (i.e. giving or receiving something from others), for example, visiting friends, having conversations and phone calls, and going to pubs. The third sphere of active ageing concerns physically or cognitively active leisure activities that the individual carries out during free time, including education, exercise and outdoor life, cultural activities, reading, and hobbies.

Like most previous studies, with some exception (for example, Kim (2019)), we do not include household activities, maintenance work, and time for personal care or caring for other members of the household in the model. Such obligations are, in this context, generally regarded as required time use. Although essential to maintain home and everyday life, they are not in focus of the third age thinking and associated active ageing concepts examined here.

## Data and methods

*Data*—We used cross-sectional Swedish Time Use Survey data to analyse how age cohorts of older adults are composed (as regards demographics and socioeconomic resources) and how they spend time on daily activities that, in line with the third age notion, may support active ageing. The data permit examination of the *young old* (65–74 years old) and *older old* (75–84 years old) in 2000/2001 and 2010/2011. We also look at changes in two consecutive pre-retirement cohorts (55–64 years old)—the *‘older middle-aged’*—seeking to identify changes among a coming generation of older people.

The data were drawn from the two most recent national time-use surveys carried out by Statistics Sweden in 2000/2001 and 2010/2011. These surveys used identical designs as regards data collection methods and instruments. The subsamples (of middle-aged and older old people) used here were representative of all people 55–84 years old registered in Sweden at the survey times and comprised a total of 712 and 920 individuals (net), in 2000/2001 and 2010/2011, respectively. In the surveys, each person kept a time-use diary for two discrete days, one weekday and one Saturday or Sunday, chosen in a random week during the year of measurement. In the diary, the day was divided into 10-min periods. For each period, respondents described in their own words what they were primarily doing. The information was then coded into over 120 main activities typical of everyday life. Coding was performed by trained staff at Statistics Sweden, using an established, detailed coding instruction manual to avoid the possibility of inter-coder discrepancies.

The response rate of the survey declined from 65% in 2000/2001 to 41% in 2010/2011, potentially creating biased results. Yet, overall comparisons between the samples and the total populations regarding known background factors indicated only small differences. Statistics Sweden assessed that the results of the survey in 2010/2011 were comparable to those of the survey conducted in 2000/2001 (Statistics Sweden, 2012: 136). However, men were somewhat overrepresented among respondents under 65 years old. Single people were slightly overrepresented in the 65–74-year age group in both 2000/2001 and 2010/2011. In the group over 75 years old, single people were slightly overrepresented in 2000/2001, but underrepresented in 2010/2011.

*Time use*—In examining overall active time use and its distribution, we operationalized the time-use classification model presented in Sect. 2. As regards the sphere of *work-related* pursuits, the data allow us to measure the time spent on: paid employment (including self-employment), caregiving for an adult or child outside one’s own household, and volunteering for formal organizations. The sphere of *social engagement* denotes time spent on: visits with relatives, friends, or neighbours; conversations and calls; visiting restaurants, cafés, bars, or pubs; and dancing and ‘partying’. The sphere of *active leisure* denotes time on: education; exercise and outdoor life; cultural activities such as going to the theatre, cinema, and shows; reading; and interests such as playing musical instruments, knitting, gardening, and gaming. In line with Kim (2019), we consider computer use cognitively active, while time-consuming TV-viewing is considered passive and non-productive.

*Sociodemographic background*—To consider structural changes in the composition of cohorts, we used selected background variables (see Table 1 in Sect. 4): respondent gender, age, and civil status (i.e. living with a partner or not); occupation (i.e. gainfully employed, retired, and other); income; and education (i.e. primary, secondary, or university level). Living regions were classified according to population density and distance to nearest city (so-called H regions). In addition, household access to a car, a computer, and housing (detached or multi-family) were included. As shown in Table 1, over the ten-year period, major shifts occurred in the composition and internal structure of the age cohorts. These are further commented on in Sect. 4, ‘Results’.

**Table 1:** Changes in sociodemographic characteristics between cohorts of the older population, Sweden, 2000/2001 and 2010/2011

	Older middle-aged (55–64 years)			Young old (65–74 years)			Older old (75–84 years)		
	2000/2001	2010/2011	Diff. 00/01–10/11	2000/2001	2010/2011	Diff. 00/01–10/11	2000/2001	2010/2011	Diff. 00/01–10/11
	*n* = 453	*n* = 444		*n* = 150	*n* = 330		*n* = 109	*n* = 146	
***Gender***									
Women	49.9%	47.3%	*–2.6%*	55.2%	52.6%	*–2.6%*	57.3%	56.3%	*–1.0%*
Men	50.1%	52.7%	*2.6%*	44.8%	47.4%	*2.6%*	42.7%	43.7%	*1.0%*
***Civil status***									
Living alone	25.9%	28.2%	*2.3%*	43.9%	28.3%	*–15.6%*	62.0%	41.9%	*–20.1%*
Cohabiting	74.1%	71.8%	*–2.3%*	56.1%	71.7%	*15.6%*	38.0%	58.1%	*20.1%*
***Age*** (years, mean)	59.0	59.7	*0.7*	68.7	68.7	*0.0*	79.1	78.7	*–0.4*
***Education***									
Primary	30.4%	18.9%	*–11.5%*	49.6%	21.6%	*–28.0%*	77.4%	43.2%	*–34.2%*
Secondary	42.5%	48.4%	*5.9%*	31.9%	47.5%	*15.6%*	13.1%	35.5%	*22.4%*
University	27.0%	32.7%	*5.7%*	18.5%	31.0%	*12.5%*	9.4%	21.3%	*11.9%*
***Occupation***									
Employed	63.7%	72.5%	*8.8%*	4.8%	7.9%	*3.1%*	0.7%	0.0%	*–0.7%*
Self-employed	10.0%	7.9%	*–2.1%*	3.1%	3.6%	*0.5%*	0.0%	0.0%	*0.0%*
Parental leave	3.5%	0.2%	*–3.3%*	0.0%	0.0%	*0.0%*	1.8%	0.0%	*–1.8%*
Student	0.4%	0.0%	*–0.4%*	0.0%	0.4%	*0.4%*	0.0%	0.0%	*0.0%*
Unemployed	3.7%	2.3%	*–1.4%*	0.3%	0.0%	*–0.3%*	0.0%	0.0%	*0.0%*
Pensioner	18.7%	17.1%	*–1.6%*	91.9%	88.1%	*–3.8%*	97.6%	100.0%	*2.4%*
***Region***									
Stockholm region	15.2%	17.5%	*2.3%*	14.5%	21.4%	*6.9%*	10.5%	16.9%	*6.4%*
Göteborg and Malmö regions	15.4%	14.4%	*–1.0%*	15.8%	10.2%	*–5.6%*	10.3%	22.5%	*12.2%*
Medium-sized city regions	36.1%	35.9%	*–0.2%*	33.1%	37.7%	*4.6%*	34.1%	26.8%	*–7.3%*
Small city regions	21.3%	17.9%	*–3.4%*	23.2%	17.0%	*–6.2%*	25.5%	23.9%	*–1.6%*
Small towns/rural regions	6.4%	6.6%	*0.2%*	3.4%	6.5%	*3.1%*	9.3%	2.1%	*–7.2%*
Remote rural areas	5.6%	7.7%	*2.1%*	10.0%	7.2%	*–2.8%*	10.2%	7.7%	*–2.5%*
***Housing***									
Single-family house	74.5%	66.2%	*–8.3%*	55.2%	66.7%	*11.5%*	54.5%	47.5%	*–7.0%*
Apartment	25.1%	33.8%	*8.7%*	44.4%	33.3%	*–11.1%*	45.5%	52.5%	*7.0%*
Income (SEK 1000, mean)	351	364	*13*	180	338	*158*	160	257	*97*
Computer access (in household)	62.8%	90.6%	*27.8%*	28.7%	82.0%	*53.3%*	7.5%	55.9%	*48.4%*
Car access (in household)	92.8%	93.2%	*0.4%*	80.1%	91.8%	*11.7%*	65.9%	77.3%	*11.4%*

Data are weighted to population totals

*Methods of analysis*—We compared how the time use of an age cohort differed from that of the corresponding age cohort ten years before (results presented in Sects. 4.1–4.3). Time use was examined by calculating the mean durations of activities separately for each comparison group. Covariate analysis (ANCOVA) was used in controlling the background factors when differences in time use between cohorts were estimated. We then employed linear regression analysis (OLS) to discern influential sociodemographic factors associated with active ageing activities at the individual level (results in Sect. 4.4). Due to small sample sizes, we then had to merge the cohorts of young old and older old into one covering the population 65–84 years old, i.e. starting from the age when most people retire in Sweden. The dependent variables were the amounts of time spent during an average day of the week on various active ageing activities.

## Results

### Cohort-wise differences in sociodemographic composition and resource availability

Table 1 shows sociodemographic and economic differences between the consecutive generations of the young old and older old occurring over the period 2000/2001 to 2010/2011. The comparison also includes the age group approaching retirement, i.e. the older middle-aged 55–64 years old. Some notable changes occurred with potential repercussions for the cohorts’ daily use of time, in turn affecting society.

In terms of demographics, the proportion of those living single declined considerably among both the young old and older old, probably due to increasing average life expectancy in Sweden. In contrast, over the years, the proportion of single middle-aged people has increased, reflecting a general trend in Swedish society. About 90% of the young old were pensioners, reflecting the fact that age 65 is the established and normally expected retirement age in Sweden. Yet, the proportion that continued to work after age 65 has been slowly increasing—a sign of active ageing and that the retirement norm is eroding. This trend might continue, given a slowly declining tendency towards early retirement (i.e. before age 65) among 55–64-year-olds. (For large parts of the Swedish labour market, there are opportunities for early retirement at age 61).

Results substantiate that newer cohorts of older people are wealthier and better educated than previous generations, establishing a foundation for a third age process. In particular, in their formative years (in the 1960s and 1970s), people belonging to the young old cohort in 2010/2011 experienced the fast expansion of the Swedish welfare state, with radically improved educational opportunities, rapidly growing incomes, improved housing and living conditions, continued urbanization, and expanding car use. In recent decades, many have also taken part in the digitalization of society. These cohort effects are reflected in the data in several ways. The proportion of people with higher education (i.e. upper secondary or university education) increased dramatically among upcoming cohorts of elders in 2010/2011, while the share of low-skilled people fell sharply. This trend continues when looking at changes among the older middle-aged, primarily in terms of the increasing proportion of graduates. Income increased the most among the young old and older old, while remaining unchanged among the middle-aged. The changing distribution of wealth between generations is further reflected in housing, as the proportion living in single-family homes increased among the young old, while decreasing among the older old and middle-aged. The change in housing relates to the slowly growing urbanization of the older population, as the proportion of older adults living in the major cities increased. As regards resources for social interaction across space, major changes occurred in car availability. Among the young old, household car access increased to a saturation level, over 90%, and in 2010/2011 equalled the level among the middle-aged. The same tendency, with some lag, was found among the older old. Digitalization, measured as having access to a computer at home, progressed very quickly. Among the recent young old, computer access increased from 29 to 82% and among the older old from 8 to 56% during the observed ten-year period.

The examination of the cohort-wise changes prompts some observations regarding the influence on the older people’s activity patterns. A first observation is that the percentage retiring at age 65 was at a high and typical level. This means that the 65–74-year age group satisfactorily represented how people with sudden access to more free time act and reorganize their everyday lives. Still, the growing number of people who continued working after age 65 indicates the increased priority given to productive activity. A second observation concerns the household and the fact that the proportion of cohabitation has been increasing among older adults, which could affect outward social activities and time use in various ways. A third observation concerns the substantial increase in access to material resources (e.g. income, housing, cars, and computers) and education among newer pensioners in general, despite the relatively short period between the surveys—ten years being a short time as regards social development. These cohort effects should have noticeable impacts on the different spheres of active ageing and related patterns of time use.

### Cohort-level differences in time use related to active ageing

Table 2 compares the amount of time the cohorts spent on different activities here associated with the concept of third age and active ageing: work activities, social engagement activities, and active leisure activities. A main observation is that participation in work activities increased among all new cohorts (in all age groups). Among the pre-retirement group of *older middle-aged* people, the increase in paid work activity (averaging 54 min per day) mainly reflected the fact that fewer chose early retirement (i.e. before age 65). Simultaneously, their engagement in the sphere of social activity diminished significantly (by 22 min per day). Also, active free-time activities such as reading books and newspapers decreased slightly (by 9 min per day), while private computer use increased (by 13 min per day).

**Table 2:** Cohort changes in time spent on activities related to active ageing between 2000/2001 and 2010/2011

Activity sphere/activity	Older middle-aged (55–64 years)			Young old (65–74 years)			Older old (75–84 years)		
	2000/01	2010/11	Diff	2000/01	2010/11	Diff	2000/01	2010/11	Diff
Work activities	265	320	54**	56	86	30*	21	46	25*
Paid work	234	294	59**	24	50	*26*	6	14	*8*
Caring for others	21	20	*–1*	21	23	*2*	7	18	11*
Voluntary work	10	6	*–4*	11	13	*2*	8	14	*6*
Social engagement activities	73	52	–22**	74	72	*–3*	68	57	*–11*
Active leisure activities	116	110	*–6*	171	177	*6*	156	195	39*
Studies	4	3	*–1*	8	5	*–3*	2	6	*4*
Exercise/outdoor activities	40	36	*–3*	55	44	*–11*	42	58	16*
Culture/entertainment	6	4	*–1*	6	11	*5*	5	8	*3*
Reading	43	33	–9*	64	57	*–7*	74	75	*2*
Hobbies	24	33	9*	37	59	21**	34	48	*14*
*Computer use*^1^	*8*	*21*	13**	*7*	*32*	25**	*2*	*15*	13**
Total, active ageing activity:	454	482	28**	301	335	34**	245	298	53**

Mean values, minutes per day (travel to and from activities included)

Significance levels: **p < 0.05, *p < 0.10

^1^Computer use (free time) is a subset of the Hobbies category

The recent *young old* cohort significantly increased their time spent in gainful employment (by 30 min per day) and computer usage (by 25 min per day) in 2010/2011 compared with the same age group in 2000/2001. Other changes were moderate, although there were signs (though not significant) of decreased time spent on active leisure activities such as physical exercise/outdoor recreation (11 min less per day) and reading (7 min less per day).

Notably, the recent *older old* cohort increased their time spent in work activities (by 25 min per day), particularly caregiving for other people, nearly as much as did the young old. They also increased their time for active leisure activities (by 39 min per day), particularly computer use (by 13 min per day) and outdoor recreation/exercise (by 16 min per day). Some reductions (though not significant) occurred in social engagement.

In sum, tendencies towards enhanced active ageing emerged in the three new cohorts in the sphere of everyday life associated with productive activities such as paid work and caregiving for others. Simultaneously, social engagement activities, comprising private interaction and bonding with other people, were declining among the older middle-aged in 2010/2011 compared with the same age group ten years before, while decreases in such activities among the young old and older old were not statistically significant. Regarding the total time spent in the active leisure activity sphere, there was a large increase among the older old, mainly due to increased outdoor recreation/exercise and computer use. The redistribution of leisure time to computer use also occurred within the two other groups—a typical period effect with huge impacts on everyday time use among the older population as well.

Overall, for all three spheres merged and over the ten years observed, there was a relatively sharp increase in active ageing activities corresponding to about 7 h per week among the older old and 3.5 h among the young old and older middle-aged.

### Controlling for differences in composition and access to resources

Still comparing an age cohort with the corresponding cohort ten years earlier, the observed differences in time use may be attributable to two circumstances: that a cohort differed in demographic and socioeconomic composition from the previous cohort, or that people prioritized their time use differently. To what extent differences in cohort-level time use depend on composition or preferences is addressed in Table 3. Here, group-level differences in mean time use are controlled statistically by taking into account variation in available compositional factors such as age, gender, civil status, income, living region, and education. Statistically significant differences in minutes used for an activity between cohorts after adjustment, indicate behavioural change, and non-significant results imply that differences in composition mainly explain the cohort-level changes in how time is spent.

**Table 3:** Estimated time use for active ageing activities (various definitions) in the cohorts of the older middle-aged, young old, and older old adults, in minutes

Activity sphere/activity	Older middle-aged (55–64 years)			Young old (65–74 years)			Older old (75–84 years)		
	2000/2001	2010 /2011	Diff	2000/2001	2010/2011	Diff	2010/2011	2010/2011	Diff
Work activities	314	382	68**	97	111	*14*	71	92	*21*
Paid work	283	360	77**	63	72	*9*	58	54	*–4*
Social activities	65	43	–22**	64	68	*4*	61	40	*–21*
Active leisure activities	100	94	*–6*	154	168	*14*	145	196	51**
Hobbies	22	30	*8*	32	59	27**	32	49	17**
Computer use^1^	7	19	12**	9	32	23**	5	17	12*

Significance levels: **p < 0.05, *p < 0.10

^1^Computer use is a subset of the Hobbies category

Concerning the pre-retirement groups (i.e. older middle-aged), results indicate that certain changes were due to both changing composition and changing priorities in the new generation. This applies to the increase in time for work, especially gainful employment, and to the decrease in social activity and the increase in leisure-time computer use. Among the post-retirement groups, preferences appear more stable, particularly regarding the young old cohorts. Here significant differences indicating behavioural change appear only in the time spent on computer use. This also holds for the members of the older old group, yet they also spent more time on active leisure activities, physical exercise, and outdoor activity in particular.

### Sociodemographic influences on active ageing

To explore at the individual level whether particular sociodemographic factors mark the active use of time, a series of multiple regressions was performed. Unfortunately, the cohort segmentation applied so far was hampered by small subsample sizes for the groups of young old and older old. Thus, the analyses here concern the entire group of *older people* 65–84 years old, i.e. starting from the age when most people retire in Sweden, while the cohort of *older middle* 55–64 years old (pre-retirement) can be kept intact. In the analyses, dependent variables are the amounts of time spent during an average day of the week on aggregated work activities, social engagement activities, and active leisure activities. Also included are results concerning the time used for substantial sub-categories: paid work, care work, voluntary work, exercise and outdoor activities, reading, and free-time computer use. Independent variables are demographic variables (i.e. chronological age, gender, and civil status), geographical variables (i.e. housing and living region), and socioeconomic resource variables (i.e. education, car access, and computer access). Car and computer access are proxies for income; cars and computers are also important tools allowing individual participation in most spheres of activity—to gain employment, engage socially, and remain active in leisure time.

As regards *older persons aged 65–84* in 2010/2011, the results indicate that a few factors had significant impacts on time use (Table 4). Within the sphere of *work activities*, time use was influenced by gender and age. Women participated to a lesser extent in such activities, their participation decreasing with increasing age. Time for paid work was also associated with civil status and education: singles worked more than cohabitants, and those with a high school education worked less than others. In contrast to the situation with paid work, women were more inclined to perform unpaid care work than were men. Participation in the sphere of *social engagement activities* was affected by gender only, with women being socially more active than men. Within the sphere of *leisure activities*, education had a significant effect, as higher education meant more active leisure time in general. Cognitively demanding reading activities were positively associated with greater age, higher education, and urban living. Regarding emerging computer use, highly educated men were more active. However, as regards physical exercise and outdoor recreation, we found no significant associations with the background factors.

**Table 4:** Regression analysis: Influence of socioeconomic factors on older individuals’ use of time related to different spheres of daily activities

	Work activities	Paid work	Care work (outside household)	Voluntary organization work	Social engagement	Active leisure	Exercise and outdoor activities	Computer use	Reading
	B	B	B	B	B	b	B	B	B
Age; 65, 66,… 84 years	–4.40***	–4.98***	–0.01	0.51	–1.33	1.79	0.71	–0.59	1.54***
Gender (male = 0, female = 1)	–25.64*	–31.25***	8.86*	–1.20	23.81***	–9.36	–2.19	–16.68***	–2.52
Civil status (single = 0, cohabiting = 1)	–14.70	–26.43*	9.89*	0.66	–11.95	13.41	11.45	2.60	–9.30
Education 1 (secondary = 0, high school = 1)	–16.37	–24.73*	3.16	1.98	0.10	19.28	–0.03	8.73	5.03
Education 2 (secondary = 0, university = 1)	0.18	–8.67	–0.91	8.54	–1.33	53.88***	2.30	16.54***	18.56**
Living region (urban = 0, rural = 1)	–5.61	1.45	–2.78	–2.48	8.11	4.69	8.14	–0.95	–11.24*
Car access (no = 0, yes = 1)	24.15	10.78	5.44	4.68	7.45	–24.69	6.26	–8.20	–3.75
Computer access (no = 0, yes = 1)	12.43	4.69	3.17	1.83	1.83	20.38	–2.64	26.83***	–4.42

Population 65–84 years old, 2010/2011 (*n* = 469)

*p < 0.10, **p < 0.05, ***p < 0.01

Fitting the same model to 2000/2001 data (see Table 5) yielded fewer significant associations than did fitting it to 2010/2011 data. At first glance, this suggests increased social and gender differences. However, due to the smaller sample size in 2000/2001, any comparison with the situation in 2010/2011 must be made with great caution. If consideration is limited to factors that were significant in 2000/2001 and that remained significant or lost significance in 2010/2010, it cannot be proven that gender or social differentiation within the spheres of active time use changed during the period. Yet, regarding specific activities, it was found that the association between gender and physical exercise and outdoor activity lost significance. Computer access ceased to be a discriminating factor regarding involvement in paid work and social engagement. Chronological age lost significance regarding care work and social engagement and remained significant in reducing time for paid work and increasing time for reading.

**Table 5:** Regression analysis: influence of socioeconomic factors on older individuals’ use of time related to different spheres of daily activities

	Work activities	Paid work	Care work (outside household)	Voluntary organization work	Social engagement	Active leisure	Exercise and outdoor activities	*Computer use*	*Reading*
	B	B	B	B	B	B	B	B	B
Age (65, 66, … 84)	− 3.27***	− 1.70*	− 1.43***	− 0.14	− 3.77**	0.66	− 1.17	0.28	1.56*
Gender (male = 0, female = 1)	− 13.57	− 11.41	− 2.89	0.74	3.72	− 18.99	− 19.51*	2.84	− 4.46
Civil status (single = 0, cohabiting = 1)	− 2.92	3.46	− 9.35	2.98	− 17.41	4.75	12.79	− 8.05	4.52
Education 1 (secondary = 0, high school = 1)	10.68	7.21	− 4.84	8.32	− 12.79	2.95	− 6.21	10.14	3.77
Education 2 (secondary = 0, university = 1)	− 0.33	− 6.75	6.38	0.05	21.07	15.91	2.02	0.17	7.37
Living region (urban = 0, rural = 1)	− 15.81	− 7.19	− 4.58	− 4.04	− 20.36	9.07	4.55	− 10.14	8.00
Car access (no = 0, yes = 1)	15.43	2.56	6.25	6.63	24.49	15.37	4.90	5.88	0.76
Computer access (no = 0, yes = 1)	37.47**	23.39*	5.19	8.89	66.57***	− 0.57	− 1.39	16.92	− 14.02

Population 65–84 years old, 2000/2001 (*n* = 257)

*p < 0.10,**p < 0.05,***p < 0.01

Overall, the results indicate that the time used for active ageing activities was not particularly socially differentiated among the large group 65–84 years old—with important exceptions concerning the differentiating roles played by gender and education. In 2010/2011, women participated to a lesser extent in paid work activities and were more inclined to perform informal care work and engage in social activities than were men. Regarding active leisure, higher education was of notable importance.

As for *the older middle-aged* cohorts, we found only a few statistically significant associations in 2010/2011 (Table 6). Paid work time and time for social engagement were negatively influenced by increasing age, whereas voluntary work was positively influenced by age and rural living. Regarding active leisure, men spent more time on computer use and women on reading. Computer use was also associated with higher education. People in rural areas spent less time reading. However, compared with the corresponding cohort ten years before, in 2000/2001 (Table 7), we found that more factors were significant at that time, signalling change. Notably, in terms of changes in social differentiation over the period, the influences of gender and education lost significance for the use of time for work and social engagement in the recent cohort of older middle-aged. Increased gender divergence was observed in the use of leisure time, as computer use became more frequent among men and reading became more frequent among women.

**Table 6:** Regression analysis: Influence of socioeconomic factors on older middle-aged individuals’ use of time related to different spheres of daily activities

	Work activities	Paid work	Care work (outside the household)	Voluntary organization work	Social engagement	Active leisure	Exercise & outdoor activities	Computer use	Reading
	B	B	B	B	B	B	B	B	B
Age (55, 56,… 64)	− 14.76***	− 16.03***	0.24	1.04*	− 14.07***	1.58	0.50	− 0.01	1.30**
Gender (male = 0, female = 1)	− 33.19	− 30.93	1.26	− 3.52	− 19.64	− 5.25	− 3.05	− 13.68***	9.90**
Civil status (single = 0, cohabiting = 1)	7.24	10.70	− 2.70	− 0.76	− 5.36	3.86	− 3.78	0.55	4.74
Education 1 (secondary = 0, high school = 1)	2.78	8.43	− 4.17	− 1.48	5.29	12.12	− 1.03	11.88*	3.31
Education 2 (secondary = 0, university = 1)	23.24	17.97	− 3.14	8.41	30.78	18.85	− 9.23	15.57**	11.11
Living region (urban = 0, rural = 1)	17.68	10.85	− 2.28	9.11**	21.41	− 8.20	− 2.98	2.25	− 9.28*
Car access (no = 0, yes = 1)	5.09	− 5.42	16.51	− 6.00	8.08	− 3.17	1.12	− 6.69	3.61
Computer access (no = 0, yes = 1)	− 0.38	9.46	− 12.61	2.77	− 1.40	24.61	10.52	21.89**	− 4.57

Population 55–64 years old, 2010/2011 (*n* = 441)

*p < 0.10, **p < 0.05,***p < 0.01

**Table 7:** Regression analysis: Influence of socioeconomic factors on older middle-aged individuals’ use of time related to different spheres of daily activities

	Work activities	Paid work	Care work (outside household)	Voluntary organization work	Social engagement	Active leisure	Exercise and outdoor activities	Computer use	Reading
	B	B	B	B	B	B	B	B	B
Age (55, 56,… 64)	− 23.68***	− 22.47***	− 0.68	− 0.53	− 24.02***	6.59***	2.48**	1.23	2.76**
Gender (male = 0, female = 1)	− 79.12***	− 83.92***	4.15	0.66	− 62.66**	6.59	0.02	1.04	4.71
Civil status (single = 0, cohabiting = 1)	− 2.31	− 4.29	9.69	− 7.71	− 19.68	3.29	10.83	− 3.58	− 6.14
Education 1 (secondary = 0, high school = 1)	− 6.95	− 4.06	3.29	− 6.17	− 8.14	24.04	11.57	2.38	5.70
Education 2 (secondary = 0, university = 1)	49.87	68.65*	− 10.90	− 7.87	48.45	34.68	14.35	7.26	6.37
Living region (urban = 0, rural = 1)	21.76	19.45	2.44	− 0.12	5.50	22.00*	9.48	− 2.34	8.52
Car access (no = 0, yes = 1)	11.08	19.31	6.80	− 15.04	30.05	16.80	15.70	− 6.72	3.36
Computer access (no = 0, yes = 1)	16.88	18.92	− 7.83	5.79	18.21	4.08	− 7.01	13.59**	3.73

Population 55–64 years old, 2000/2001 (*n* = 454)

*p < 0.10, **p < 0.05, ***p < 0.01

## Concluding discussion

In this paper, we explore differences in active ageing activities among consecutive cohorts of older adults in Sweden by scrutinizing the reorganization of their daily use of time over a period of ten years. The results contribute to the wider discussion of evolving activity patterns, with implications for well-being and health in ageing societies. Drawing on the expectations of third age thinking (Laslett 1989, 1994) and the associated active ageing framework (e.g. Foster and Walker 2014; WHO 2015; Zaidi and Howse 2017), we found that new generations are generally more active in terms of their time use than were previous ones. Sizeable increases in overall active time use were observed among newer cohorts of the pre-retirement middle-aged, young old, and older old. Yet, since the concept of active ageing is inconsistently defined in current research (Boudiny 2013), we refined the analysis to discern underlying tendencies as regards specific types of activity. For this purpose, we developed a conceptual model of active ageing time use associated with different spheres of everyday life. We could then discern several tendencies, both reinforcing and compensating for time use relationships.

First, in all age groups, we found a significant increase in the sphere of *work activities*, that is, time spent producing (paid or unpaid) goods and services for society. Among the older old, caregiving work was on the rise. Among the young old, there was a significant increase in paid working time, indicating that new generations are more likely to remain in active employment after the hitherto standard age of retirement. Also, the older middle-aged have become less inclined to choose early retirement. Thus, our findings stand in contrast to tendencies towards early retirement and lowered working hours among the new generations of older people reported in previous research (Gauthier and Smeeding 2010; Jun and Suhs 2019). From an active ageing perspective, our findings are supportive, as they indicate that the older population is increasingly remaining productive and contributing to society. Yet, from an individual health and well-being perspective, we need to understand how this tendency relates to the other spheres of active ageing.

Second, and countering the third age expectations, we found decreases in time spent in the *social engagement sphere* of everyday life, comprising social activities within personal networks. Decline in social activity was discerned in the pre-retirement group and weakly indicated in the other groups, and is a trend also observed in a recent time-use study in the UK (Jun and Suhs 2019). Since social engagement is vital to individual health and well-being, this issue deserves further research, for example, to determine whether the decline in social activity found among the older middle-aged cohort will continue after retirement. Also, there is a need to deepen our understanding of how, and in what contexts, loneliness occurs and develops in later life, for example, whether activities are increasingly performed alone, at home or in public spaces, and to what extent access to space-transcending technologies, such as cars, public transportation, and the Internet, encourage everyday sociality (Thulin and Vilhelmson 2019).

Third, important changes supportive of the third age concept were also found in the more individually centred *sphere of active leisure time*. In particular, active leisure engagement has increased in the older old group, who devote more time to outdoor activity and exercise, probably with positive health implications. Crucial changes are further linked to the digitalization of society, as preferences and time for computer use are increasing substantially in all age groups. Though this trend is expected, it is ambiguous from an active ageing perspective focusing on individual health and well-being (Thulin and Vilhelmson 2019). On one hand, computer use enables virtual social participation and contact that facilitates an active lifestyle and leisure time. On the other hand, it is a sedentary screen activity, parallel to TV watching (in the literature often considered the ultimate passive activity), which does not fit well with the goals of active ageing. To fully understand the implications of digitalization for the daily activities, well-being, and health of old people, more detailed research on their time online is needed, for example, regarding how online activity interacts with the other spheres of activity in everyday life.

Fourth, our study indicates that the observed differences in the older individual’s use of time in wide-ranging active spheres were largely attributable to changes in the socioeconomic and demographic composition of the cohorts. However, there are some indications of changing preferences in activity choice and time use. Behavioural changes particularly concern the increase in paid work and the decrease in social activity among the new cohort of people in the pre-retirement phase, as well as the tendency of upcoming older old people to spend more time on active leisure activities. In all groups, a tendency to prioritize more time at the computer was observed.

Fifth, there are reasons to believe that the main tendencies discussed here are socially differentiated. It is a common criticism of the third age concept that it risks treating older generations as homogenous, comprising active and well-off people (e.g. Lassen and Moreira 2014; Wanka 2020). Our cross-sectional regression analyses of people in the post-retirement period (i.e. 65–84 years old) indicate that women (vs. men) spent a little less time on paid work and more time on social activity, pointing to the gendered aspects of active ageing (as emphasized by, e.g. Foster and Walker 2013). Furthermore, higher education was positively associated with active leisure time, also corroborating the findings of other recent studies (Sabbath et al. 2016; Kim 2019) and pointing to inequalities in current ageing. However, when looking at changes occurring between the cohorts 65–84 years old, we could not find signs of enhanced inequality in time use for active ageing activities, although this observation should be treated with some caution due to the small sample size. Also, between the cohorts aged 55–64 years, next in line to enter old age and retirement, the crucial factors of gender and education lose significance over time. Arguably and looking ahead, this indicates that the main tendencies in active ageing time use discerned in this study will gradually come to include broader groups of society.

The study has some limitations. The data do not include direct information on individual wealth (e.g. income and housing) and social networks (e.g. children and close friends), both of which have documented positive effects on health and activity in old age. Nor is there information on occupational history, preventing us from identifying the negative impacts of having experienced a strenuous working life. This means that the distributional aspects of work life, social ties, and material wealth are probably underestimated. The results might also be affected by some sample bias, as women were marginally underrepresented among the older middle-aged cohorts, and the non-response rates fluctuated slightly regarding single status among the post-retirement groups.

Furthermore, our results entail limitations as they do not include data from the most recent years. It is therefore important to point out that the results capture slow-moving structural processes, and that the observed trends mainly tended in same direction among the three age cohorts under study. This also means that the observed shifts in time use are likely to be reactivated after disruptive events, such as the current Covid pandemic, and when forced limitations in older people's activity spaces are eased. Still, our findings stress the importance of continuously examining the diverse lifestyles of upcoming cohorts of older adults—i.e. their activity patterns in terms of everyday time use—and the implications of these for society and policymaking. Some of the trends identified here have probably intensified in recent years. A look at the older middle-aged cohort in 2010/2011 illustrates how their situation might appear in a contemporary context. Compared with the young old and the older old in 2010/2011, the middle-aged were more educated, somewhat more gender equal, and had greater experience with computers, social media, and cars. Presumably, they were far more influenced by the social norms and expectations of active ageing (e.g. articulated by a higher legal retirement age). Most certainly, they entered ‘old age’ with even higher levels of work time engagement than did previous generations, spending more time using computers, mobile digital media, and cars. Yet, following the trends observed in this group, we might also expect less involvement in the sphere of social engagement. Thus, in coming years it will be even more important, from a health perspective, to follow up on, evaluate, and influence time-use changes, and to further consider the associated social differentiation of active ageing.

The inequality aspect also calls for critical consideration of the ‘active’ activity concept in the context of time use and well-being. For example, increases in time for work activity can be driven not only by the free choices of active ‘third agers’, but also by the needs of people who cannot afford to retire due to lack of resources and reduced pensions. Also, the increased time the oldest cohort spent caregiving for others might be a response to ongoing shifts in the provision of elderly care in Sweden, from institutional care to mobile home-help services (Larsson et al. 2006). Home help facilitates the everyday life of older people, enabling them to stay in their own homes for as long as possible, but might simultaneously increase stress on and demands for assistance from spouses and close relatives.

Furthermore, from a health perspective, we need to improve our understanding of the relationships between different spheres of active ageing, given that daily time is a limited resource. Time spent on different ‘active’ activities competes with and displaces ‘passive’ activities within the 24 h of the day (Thulin and Vilhelmson 2019). Notably, our findings indicate that work commitment may displace engagement in social activities, possibly with negative implications for individual health and well-being. Increased caring obligations may take time from active leisure and severely constrain the choice of daily activities both in and out of the home. More sedentary time spent on computers and digital media screens may compete with and counteract the trend towards active leisure pursuits among older people as well, for example, replacing time spent with friends or grandchildren. For future research, this emphasizes a need to better understand the contextually dependent nature of the active/passive activity distinction, which is becoming particularly critical in the period coming after the third age. As explained by Clarke and Warren (2007:465), in later life ‘even stopping paid work and entering residential care may be actively chosen and empowering even though they are steps towards disengagement and dependency’.

## References

[CR3] Atchley RC (2001). Continuity and adaptation in aging: creating positive experiences.

[CR4] Baltes PB, Smith J (2003). New frontiers in the future of aging: from successful aging of the young old to the dilemmas of the fourth age. Gerontology.

[CR5] Bass S (2000). Emergence of the third age: toward a productive aging society. J Aging Soc Pol.

[CR6] Bass S, Caro FJ, Chen Y-P (1993). Toward a productive aging society.

[CR7] Bauman A, Bittman M, Gershuny J (2019). A short history of time use research; implications for public health. BMC Public Health.

[CR8] Bell A (2020). Age period cohort analysis: a review of what we should and shouldn’t do. Ann Hum Biol.

[CR9] Boudiny K (2013). ‘Active ageing’: from empty rhetoric to effective policy tool. Ageing Soc.

[CR10] Boudiny K, Mortelmans D (2011). A critical perspective: towards a broader understanding of ‘active ageing’. Electron J Appl Psychol.

[CR11] Butler RN, Gleason HP (1985). Productive aging: enhancing vitality in later life.

[CR12] Casalanti TM (1996). Incorporating diversity, levels of research, and implications for theory. Gerontologist.

[CR13] Chatzitheochari S, Arber S (2011). Identifying the third agers: an analysis of british retirees' leisure pursuits. Sociol Res Online.

[CR14] Clarke A, Warren L (2007). Hopes, fears and expectations about the future: what do older people’s stories tell us about active ageing?. Ageing Soc.

[CR15] Cumming E, Dean LR, Newell DS, McCaffrey I (1960). Disengagement – a tentative theory of aging. Sociometry.

[CR16] Foster L, Walker A (2013). Gender and active ageing in Europe. Eur J Ageing.

[CR17] Foster L, Walker A (2014). Active and successful aging: a European policy perspective. Gerontologist.

[CR18] Gauthier AH, Smeeding TM (2003). Time use at older ages cross-national differences. Res Aging.

[CR19] Gauthier AH, Smeeding TM, Tuljapurkar S, Ogawa S, Gauthier AH (2010). Historical trends in the patterns of time use of older adults. Aging in advanced industrial states.

[CR20] Gershuny J (2000). Changing times. Work and leisure in postindustrial society.

[CR21] Gilleard and Higgs (2002). The third age: class, cohort or generation?. Ageing Soc.

[CR22] Gonzales E, Matz-Costa C, Morrow-Howell N (2015). Increasing opportunities for the productive engagement of older adults: a response to population aging. Gerontologist.

[CR23] Havighurst RJ (1961). Successful aging. Gerontologist.

[CR24] Hinterlong J, Morrow-Howell N, Sherraden M, Morrow-Howell N, Hinterlong J, Sherraden M (2001). Productive aging: principles and perspectives. Productive aging: concepts and challenges.

[CR25] Hochschild AT (1975). Disengagement theory: a critique and proposal. Am Soc Rev.

[CR26] Huang W (2018). The social determinants of activity pattern among older adults: a latent profile analysis on time use data. Innov Aging.

[CR27] Hunt E, McKay, (2015). A scoping review of time-use research in occupational therapy and occupational science. Scand J Occup Therapy.

[CR28] Jun J, Suhs J (2019) Time use and wellbeing in later life. In: Gershuny J, Sullivan O (eds) What we really do all day. Insight from the center for time use research*.* pp 265*–288*

[CR29] Katz S (2000). Busy bodies: activity, aging and the management of everyday life. J Aging Stud.

[CR30] Kim JH (2019). Productive aging of Korean older people based on time use. Soc Sci Med.

[CR31] Larsson K, Thorslund M, Kåreholt I (2006). Are public care and service for older people targeted to need? Applying the behavioural model on longitudinal data of a Swedish urban older population. Eur J Ageing.

[CR32] Laslett P (1989). A fresh map of life: the emergence of the third age.

[CR33] Laslett P (1994). The third age, the fourth age and the future. Ageing Soc.

[CR34] Lassen AJ, Moreira T (2014). Unmaking old age: political and cognitive formats of active ageing. J Aging Stud.

[CR35] Marcum CS (2013). Age differences in daily social activities. Res Aging.

[CR36] Michelson W (2015). Time use: expanding explanation in social sciences.

[CR37] Morrow-Howell N, Hinterlong J, Sherraden M (2012). Productive aging: concepts and challenges.

[CR38] Morrow-Howell N, Gehlert S (2102) Social engagement and a healthy aging society. In: Prohaska T, Anderson L, Binstock R (eds) Public health for an aging society*,* The Johns Hopkins University Press, Baltimore, 212–227

[CR39] Nilsson K (2016). Conceptualisation of ageing in relation to factors of importance for extending working life – a review. Scandinavian Journal of Public Health.

[CR40] Principi A, Santini S, Socci M, Smeaton D, Cahill KE, Vegeris S, Barnes H (2018). Retirement plans and active aging: perspective in three countries. Ageing Soc.

[CR41] Rowe JW, Kahn RL (1997). Successful aging. Gerontologist.

[CR42] Rowe JW, Kahn RL (2015). Successful aging 2 0: Conceptual expansions for the 21st century. J Gerontol B Psychol Sci Soc Sci.

[CR43] Sabbath EL, Matz-Costa C, Rowe JW, Leclerc A, Zins M, Goldberg M, Berkman LF (2016). Social predictors of active life engagement: a time-use study of young-old french adults. Res Aging.

[CR44] Schwanen T, Ziegler F (2011). Wellbeing, independence and mobility: an introduction. Ageing Soc.

[CR45] Shove E (2009) Everyday practice and the production and consumption of time. In: Shove et al (Eds) Time, consumption and everyday life. practice, materiality and culture, pp 17–33, Berg, New York.

[CR46] Skoog, I. (2020) 70 är det nya 50. Rapport 21. Delegationen för senior arbetskraft. S 2018:10. Stockholm.

[CR47] Sprod JA, Ferrar K, Olds T, Maher C (2015). Changes in sedentary behaviours across the retirement transition: a systematic review. Age Ageing.

[CR48] Sprod J, Olds T, Brown W, Burton N, van Uffelen J, Ferrar K, Maher C (2017). Changes in use of time across retirement: a longitudinal study. Maturitas.

[CR100] Thulin E, Vilhelmson B (2019) More at home, more alone? Youth, digital media and the everyday use of time and space. Geoforum 100:41–50

[CR2] Vilhelmson B, Thulin E, Elldér E (2017) Where does time spent on the internet come from? Tracing the influence of information and communications technology use on daily activities. Inf Commun Soc 20(2):250–263

[CR49] Walker A (2002). A strategy for active ageing. Int Soc Secur Rev.

[CR50] Walker A (2009). The emergence of active ageing in Europe. J Ageing Soc Policy.

[CR51] Walker A, Zaidi A (2016). New evidence on active ageing in Europe. Intereconomics.

[CR52] Wanka A (2019) No time to waste. How the social practices of temporal organization change in the transition from work to retirement, Time Soc, 494–517

[CR53] Wanka A (2019). Change ahead – emerging life-course transitions as practical accomplishments of growing older. Front Sociol.

[CR54] Wanka A (2020). Continuity and change in the transition to retirement: how time allocation, leisure practices and lifestyles evolve when work vanishes in later life. Eur J Ageing.

[CR55] Weir DR, Waite LJ, Wong R, Freedman VA, Hayward MD, Majmundar MK (2018). New measures and new designs in demography of aging research. Future directions for the demography of aging.

[CR56] WHO (1990). Healthy ageing, technical report.

[CR57] WHO (2002). Active ageing: a policy framework.

[CR58] WHO (2015). World report on ageing and health.

[CR59] Zaidi A, Howse K (2017). The policy discourse of active ageing: some reflections. Population Ageing.

